# Medical Research Council dyspnea scale does not relate to fibroblast foci profusion in IPF

**DOI:** 10.1186/1746-1596-6-28

**Published:** 2011-04-05

**Authors:** Christina Triantafillidou, Effrosyni D Manali, Christina Magkou, Christina Sotiropoulou, Likurgos F Kolilekas, Konstantinos Kagouridis, Dimitra Rontogianni, Spyros A Papiris

**Affiliations:** 1 2nd Pulmonary Department, "Attikon" University Hospital, Athens Medical School, National and Kapodistrian University of Athens, Greece; 2 "Sotiria" General Hospital, Athens Greece; 3 Department of Pathology, "Evangelismos" General Hospital, Athens, Greece; 4 Applied Biomedical Research & Training Center "Marianthi Simou" and 1st Department of Critical Care & Pulmonary Services, School of Medicine, National and Kapodistrian University of Athens, Greece

**Keywords:** dyspnea, pulmonary fibrosis, fibroblast foci

## Abstract

**Background:**

In Idiopathic pulmonary fibrosis (IPF) irreversibly progressive fibrosing parenchymal damage, leads to defects in mechanics and gas exchange, manifesting with disabling exertional dyspnea. Previous studies have shown a relationship between fibroblast foci (FF) profusion and severity and survival and a relationship between dyspnea grade and severity and outcome. We hypothesized a relationship between Medical Research Council (MRC) dyspnea scale with FF, and a relationship between FF and functional parameters and survival.

**Methods:**

We retrospectively reviewed 24 histologically documented IPF patients. Profusion of FF was semiquantitatively evaluated by two scores, Brompton and Michigan. Survival analysis was performed by fitting Cox regression models to examine the relationship of the two scores with survival and the non-parametric Spearman correlation coefficient was calculated to describe the relationships of FF scores with dyspnea scores and functional parameters.

**Results:**

No statistically significant correlation between FF scores and the MRC scores was observed (p = 0.96 and p = 0.508 respectively). No significant correlation between FF scores and survival (p = 0.438 and p = 0.861 respectively) or any functional parameter was observed.

**Conclusions:**

The lack of relationship between the MRC dyspnea scale and the FF might relate to the fact that dyspnea in IPF better reflects the overall of lung damage and its related consequences on mechanics and gas exchange whereas FF, one of its histological hallmarks, may not reflect its entire histology derangement also constrained by the geographically limited sampled tissue. This might be also valid for the observed lack of association between FF and survival or functional parameters.

## Background

Idiopathic Pulmonary fibrosis (IPF) is a dreadful and incurable, chronic and irreversibly progressive fibrosing lung disease [1]. Inflammation and fibrosis constitute the mainstay of parenchymal lung damage. Progression of lung damage leads to defects in mechanics and gas exchange and clinically manifests with progressive exertional dyspnea leading to disability, ending to death.

The diagnostic histopathologic features of usual interstitial pneumonia (UIP) consist at low-magnification view of a patchwork pattern of lung involvement with honeycomb areas alternating with normal lung and parenchymal scarring and at higher magnification of inflammation overshadowed by fibrosis and polyclonal fibroblastic aggregates called fibroblast foci (FF) coexisting with areas of inactive collagen-type scarring [2]. Fibroblast foci are considered foci of active, currently ongoing interstitial fibrosis. The above histological pattern is valuable for diagnosis in the non typical cases since according to several studies the diagnosis of UIP/IPF can be made with confidence based on clinical and roentgenographic findings in the most (typical) cases [1,3-5].

The clinician caring for IPF patients necessitates non-invasive, simple, reliable and reproducible parameters to estimate the severity, progression and prognosis of the disease. Regarding clinical parameters, several studies have shown that different scores of exertional dyspnea constitute a reliable tool which serves the function described above [6-11]. This is also the case for functional parameters such as the total lung capacity (TLC), the forced vital capacity (FVC), the carbon monoxide diffusing capacity (DLCO) [12,13] and their deterioration over time [14], the desaturation and the distance walked during the six-minute walk test [15,16] and the PaO_2_-slope and the maximal oxygen uptake during cardiopulmonary exercise testing [17,18]. Last but not least among the non-invasive parameters evaluating IPF is the extent of fibrosis on high-resolution computed tomography scans [6,19].

Further studies examining the relationship between histopathology and survival of patients with IPF, have highlighted the potential role of the profusion of FF as predictor of survival in IPF patients. However, results have been controversial so far with investigators concluding either "pro" or "con" their prognostic significance [20-26]. Although progressive dyspnea is the most prominent symptom in UIP/IPF patients, the relationship between dyspnea and histopathologic features of disease such as fibroblastic foci, is not studied. In this study we hypothesized a relationship between the Medical Research Council (MRC) chronic dyspnea scale and the FF profusion in surgical lung biopsies from UIP/IPF patients at diagnosis, as well as a relationship between FF and functional parameters of disease severity and survival.

## Methods

### Subjects

This study was approved by the "Scientific Board-IRB-Committee of Bioethics, Attikon University Hospital Athens Greece", National and Kapodistrian University of Athens. Patients fulfilling the criteria of the American Thoracic Society and European Respiratory Society for the diagnosis of IPF [27] and the histological pattern of UIP on surgical lung biopsy obtained through open thoracotomy or video-assisted thoracoscopy performed from February 1998 through December 2006 were included retrospectively (clinical information was obtained from medical records). Αll patients were treatment naive when surgical lung biopsy was performed. Secondary causes of lung fibrosis were excluded: none of the patients had a history of environmental or occupational exposure, drug toxicity or autoimmune rheumatic disease, as documented by history, clinical and immunological tests. Survival time was calculated from the time of surgical lung biopsy until their death or time of verification of vital status, which was ascertained by follow-up with the subjects directly, their families or personal physicians.

### Dyspnea

Dyspnea was assessed at diagnosis by the treating physicians using the modified MRC chronic dyspnea self-administered questionnaire consisting of six questions about perceived breathlessness: 0, no dyspnea; 1, slight dyspnea (shortness of breath when hurrying on the level or walking up a slight hill); 2, moderate dyspnea (walks slower than people of the same age on the level because of breathlessness); 3, moderately severe dyspnea (stops because of breathlessness when walking at own pace on the level); 4, severe dyspnea (stops for breath after walking about 100 yards or after a few minutes on the level); 5, very severe dyspnea (too breathless to leave the house or breathless when dressing or undressing) [7].

### Pulmonary Functional Tests (PFTs)

PFTs were done at diagnosis at an interval not more than a month prior to biopsy. PFTs included forced expiratory volume during the first second of expiration (FEV_1_), forced vital capacity (FVC), total lung capacity (TLC), and single-breath carbon monoxide diffusing capacity (DLCO) all measured by MasterScreen Body apparatus (Erich Jaeger GmbH, Wuerzburg, Germany). Measurements are expressed as absolute values and as percent of predicted normal.

### Pathologic assessment

**Figure 1:**
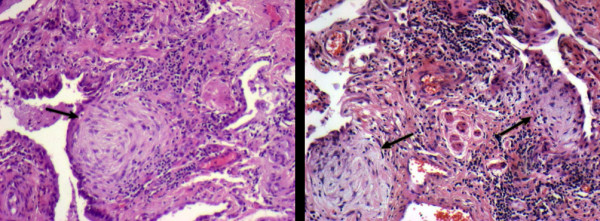
**High magnification view stained with hematoxylin-eosin showing areas of fibroblast foci (arrows)**.

Lung specimens were obtained from at least two lobes of the same lung, when technically feasible. All slides were reviewed separately and without knowledge of the clinical data by two independent pathologists (CM, DR). Both pathologists were unaware of the clinical and functional characteristics of the patients. Patients whose histopathologic findings were not consistent with UIP were excluded. Ten randomly selected non-overlapping fields stained with hematoxylin-eosin were viewed at low-power magnification (x40) and scored semiquantitatively for profusion of FF (Figure 1) by two different score systems: the Brompton score, using a 0-6 scale (0 represents an absence of FF, while a 6 or more FF in the examined field is scored as 6) and the Michigan score, using a 0-3 scale (0: absent, 1: mild, 2: moderate, 3: marked) as previously described [21,22]. In cases with biopsies from two different sites, an average score was calculated. To evaluate the lymphocyte CD8 subpopulation lung tissues were stained with mouse monoclonal antibodies anti-pan-T cell (anti CD3; dilution, 1:200) and anti CD8 (dilution 1:40) [Dako; Glostrup, Denmark] according to the labelled streptavidin-biotin complex method. The sections were deparaffinized and rehydrated with Tris-buffered saline solution (0.005 mmol/L Tris and 0.15 mmol/L NaCl; pH, 7.6) for 10 min. Endogenous peroxidase was blocked with 3% hydrogen peroxide for 5 min. Then the sections were washed in Tris-buffered saline solution and incubated with primary antibodies at appropriate dilutions for 1 h. Biotinylated antimouse IgG was used as a secondary antibody (Dako), followed by peroxidase-conjugated streptavidin (Dako). The peroxidase reaction was developed using 3,3'-diaminebezidine tetrachloride (0.25 mg dissolved in 1 mL of 0.02% hydrogen peroxide) for 3 min. The results were expressed as the percentage of nuclear immunopositive surface in relation to the total nuclear surface of infiltrative cells within the tissue.

### Statistical analysis

Data are presented as mean ± SD or median (range) values. Mean values of FF scores of the two observers were used in the statistical analysis. Inter-rater agreement was estimated by the weighted kappa statistic. The non-parametric Spearman correlation coefficient was calculated to describe the relationships of Brompton and Michigan scores with the other examined variables. Survival analysis was also performed by fitting Cox regression models to examine the relationship of the two scores and MRC with survival. The Kaplan-Meier method was used to estimate survival distribution. The two scores and the MRC were also divided into two groups using their median value and comparison of their survival distributions were made by the log-rank test. Statistical analysis was performed by the Statistical Package for the Social Sciences Software Version 11.0 (SPSS, Chicago, IL).

## Results

The population studied consisted of 24 patients with clinical, radiological and pathological features of IPF. The demographic, clinical, and functional characteristics of the study population at the time of diagnosis are given in Table 1. Eleven patients were female and thirteen were male with a mean age of 63.5 ± 7 years. Half of the patients were no smokers, the rest being ex-smokers. All patients were treatment naïve at the time of surgical biopsy. After entry into the study, 18 patients received treatment with immunosuppressive or cytotoxic therapy (low dose prednisolone alone or in combination with azathioprine, or mycophenolate mofetil or colchicine, withdrawn and not substituted at first sign of opportunistic infection from the lungs or other organs), while 6 patients never received any treatment. Nine patients had an MRC score of 1, ten a score of 2, three a score of 3 and two a score of 4. No patient included in the study had an MRC score of 5. The results of the PFTs are shown in Table 1. All patients had a restrictive pattern with a mean value for FEV_1_/FVC ratio of 88 ± 5.6%, for TLC% of 61.7 ± 11.7% and 43.6 ± 14.4% for DLCO%. Multiple biopsies were performed in the majority of patients (18 out of 24, 75%). The profusion of FF in lung biopsy specimens according to the Brompton and Michigan scores was found at a median value of 2,2 and 1,46 respectively (Table 2).

**Table 1:** Demographic, clinical and functional data of the study population (n = 24)

**Age, year (mean ± SD)**	63.5 ± 7
**Sex (M/F)**	13/11
**Alive/Dead**	16/8
**Smoking history (n %)**	
**Ex smokers**	12 (50%)
**No smokers**	12 (50%)
**Current smokers**	0 (0%)
**MRC chronic dyspnea score, n (%)**	
0	0 (0%)
1	9 (37,5%)
2	10 (41,7%)
3	3 (12,5%)
4	2 (8,3%)
5	0 (0%)
**PFTs (mean ± SD)**	
FEV_1 _- FEV_1 _% pr	1,97 ± 0,4 - 83,7 ± 15,9%
FVC - FVC % pr	2,25 ± 0,6 - 76,2 ± 15,2%
FEV_1_/FVC (ratio) % pr	88 ± 5,6%
TLC - TLC % pr	3,31 ± 0,7 - 61,7 ± 11,7
DLCO - DLCO % pr	3,23 ± 1,2 - 43,6 ± 14,4%

M/F Male/Female, FEV_1 _Forced expiratory volume at 1 second, % per cent, FVC Forced vital capacity, TLC Total Lung Capacity, DLCO Diffusion capacity for carbon monoxide, FEV_1_/FVC: FEV_1_/FVC ratio

**Table 2:** The profusion of fibroblast foci according to two different scales (N = 24)

**Brompton score**	**N**	**(%)**
0	0	0
1	7	29.2
2	10	41.7
3	3	12.5
4	3	12.5
5	1	4.2
6	0	0
Brompton score	**Median **2,2	**Range **(1, 5)
**Michigan score**	**N**	**(%)**
0	0	0
1	15	62.5
2	7	29.2
3	2	8.3
Michigan score	**Median **1,5	**Range **(1, 3)

**Figure 2:**
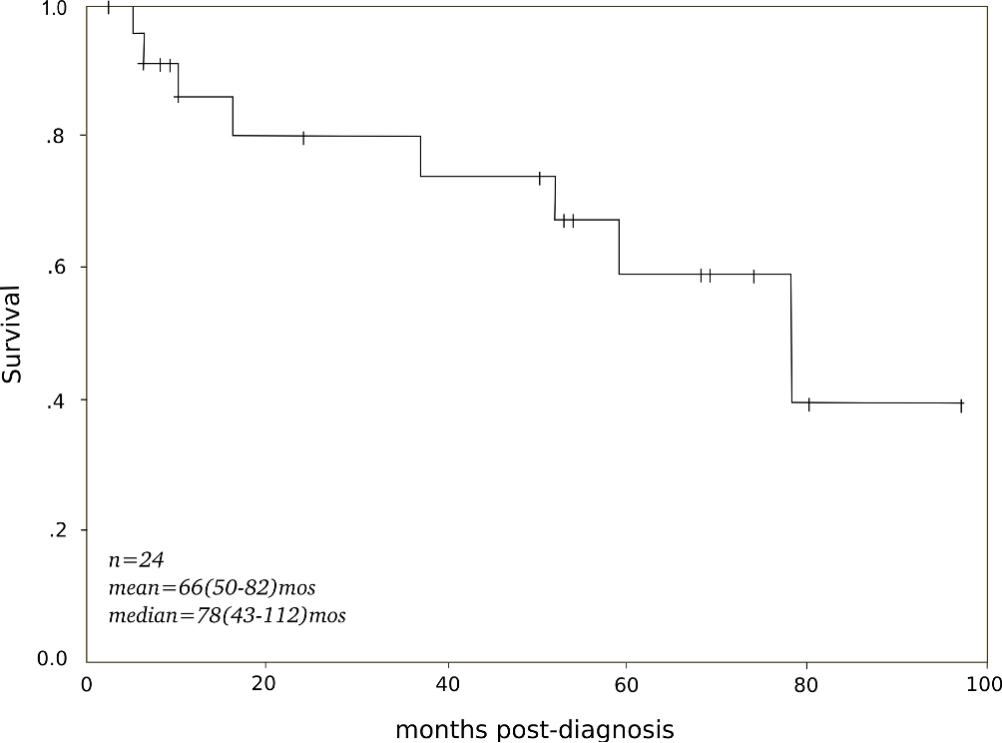
**The cumulative Kaplan-Meier survival plot**. Survival of 24 patients with histologically documented UIP/IPF, followed till death (uncensored: n = 8) or reporting of the study (censored: n = 16). Shown are sample size (n) and survival [mean and median (95% Confidence Interval)].

At the time of reporting of this study, eight patients had succumbed to IPF while sixteen patients were still alive (Figure 2). Follow-up time ranged from 2 months to 8.08 years. Median survival was 78 months (95% CI 43.66-112.34). All deaths were directly attributable to the disease, a fact verified by death certificates. Interobserver agreement for histopathologic scores was substantial for both scoring systems (Brompton score Weighted Kappa = 0.6, Michigan score Weighted Kappa = 0.7). No significant correlation was found between both Brompton or Michigan score and the MRC dyspnea score (r = -0.348, p = 0.96 for Brompton score, r = -0.142, p = 0.508 for Michigan score) (Table 3).

**Table 3:** Relationships between the fibroblast foci scores and the other examined parameters in the study population (N = 24)

Variables		Brompton score	Michigan score
**mMRC**	r	-0.348	-0.142
	p-value	0.096	0.508
**FEV**_**1 **_**%**	r	0.122	-0.086
	p-value	0.570	0.688
**FVC %**	r	0.010	-0.157
	p-value	0.964	0.463
**FEV**_**1**_**/FVC %**	r	0.172	0.063
	p-value	0.422	0.770
**TLC %**	r	-0.170	-0.118
	p-value	0.426	0.584
**DLCO %**	r	0.205	0.090
	p-value	0.337	0.676

The non-parametric Spearman correlation coefficient was used. FEV_1 _Forced expiratory volume at 1 second, % per cent, FVC Forced vital capacity, TLC Total Lung Capacity, DLCO Diffusion capacity for carbon monoxide, FEV_1_/FVC: FEV_1_/FVC ratio

**Figure 3:**
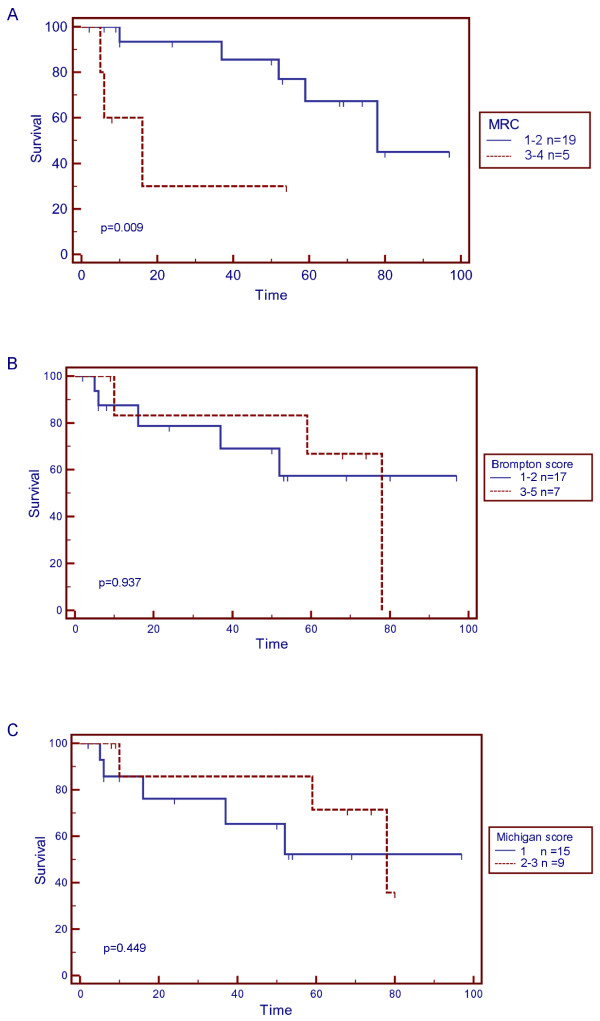
**Twenty four patients with histologically documented UIP/IPF were followed till death (uncensored n = 8) or reporting of the study (censored n = 16)**. (A) Kaplan-Meier survival curve for MRC is shown. Patients with higher MRC scores experienced shorter survival (p = 0.009). Kaplan-Meier survival curves for both Brompton (B) and Michigan (C) scores are shown. No significant correlation was found between the two subgroups and survival.

Furthermore, no significant correlation was documented between FF scores (both Brompton and Michigan score) and survival (Cox regression results: a) Brompton score: RR = 1.234, p = 0.438, 95% CI = 0.725-2.102, b) Michigan score: RR = 1.102, p = 0.861, 95% CI = 0.372-3.269, or any of the other examined variables of the pulmonary function tests (Table 3). However, the MRC dyspnea score was found significantly correlated with survival (RR = 2.283, p-value = 0.020, 95% CI: 1.137-4.582, n = 24). Kaplan-Meier curves for MRC and FF scores are shown in Figure 3 and OR with CIs were as follows: OR for MRC: group 0-2, group 3-5: p = 0.17, OR = 4.2, 95% CI = 0.535-32.956, OR for Brompton score group 1-2, group 3-5: p = 0.5, OR = 1.8, 95% CI = 0.290-11.161, ΟR for Michigan score: group 0-1, group 2-3: p = 1, OR = 1, 95% CI = 0.173-5.772.

As CD8 was another histopathologic feature of IPF biopsies found to correlate with MRC in recent studies of our group [7], the correlation of tissue CD8 with both MRC scale and survival was examined with no statistically significant relationships found: (r = 0.154, p = 0.555, RR = 1.03, p = 0.42, 95% CI = 0.95-1.11, n = 17 respectively)

## Discussion

In this study we hypothesized a relationship between the MRC chronic dyspnea scale and the FF profusion scores in surgical lung biopsies from UIP/IPF patients at diagnosis, to the best of our knowledge for the first time. In addition we hypothesized a relationship between FF and functional parameters of disease severity and survival in our population studied. No statistically significant correlation between FF scores and the MRC score was observed. No significant correlation between FF scores and functional parameters of disease severity as well as survival was documented.

Previous studies have shown that the MRC scale, a non-invasive and simple clinical tool to estimate chronic dyspnea, is reliable in the estimation of the severity, progression and prognosis in IPF patients [7,8,10]. Furthermore, previous studies of our group detected also a relationship between the MRC scale and tissue infiltrating CD8+ T lymphocytes as well as CD8+ T lymphocytes recovered by bronchoalveolar lavage [28,29] as well as a relationship between the MRC dyspnea and physiological parameters obtained during maximal and submaximal exercise testing known to reflect exercise limitation, disease severity and survival in IPF [30]. Therefore, we assumed that the MRC score could also be related with the FF profusion score in IPF lung biopsies, which has been shown predictive of survival in previous studies [20,21,23,24]. Nevertheless, the results of the current study do not support our hypothesis of a relation between this histopathologic characteristic of IPF and the degree of dyspnea.

To the best of our knowledge there is only one study examining the relationship between worsening dyspnea over time (6 months) and the FF profusion in pharmacologically treated UIP/IPF patients that apparently contradicts our results [25]. However, in this study Collard and co-workers used a different dyspnea scale extending from 0 (no dyspnea) to 20 (dyspnea at rest) and the relationship between dyspnea and the FF profusion at initial evaluation of patients and before treatment initiation is not reported.

Regarding our second endpoint, no significant association was found between survival and the profusion of FF in surgical lung biopsies from patients with IPF, measured semiquantitatively by the two already described score systems: the Brompton and the Michigan score [21,22]. Published studies show contradictory results: in agreement to our results, Flaherty and co-workers did not find the profusion of FF to be a significant predictor of survival for 99 patients with UIP by any method (the Michigan score, the Brompton score and the Denver score). Accordingly, Collard and co-workers did not find a relationship between survival and any individual histopathological feature, including the profusion of FF [25]. Similarly, in a recent work Hanak and co-workers failed to detect any relationship between FF prevalence and survival in IPF patients [26]. In contrast to the above, other investigators succeeded to detect a relationship between different methodologically obtained scores for the estimation of the FF profusion and survival [20,21,23,24].

As far as the association between the profusion of FF and functional parameters is concerned, our results are similar to the ones of Enomoto and co-workers [23]. The investigators who found a relationship between the extent of FF and functional parameters mostly examined changes of functional parameters over time such as the decrease in both DLCO and FVC measured at 6 and 12 months after biopsy [21] and the worsening in percent predicted FVC over 6 months of follow up [25]. That is, the profusion of FF was not correlated with the initial pulmonary function status, but with their deterioration over time.

In order to explain these contradictory results, one notices following a critical and comparative analysis of reported studies differences that mainly concern the length of survival and the methods of assessing the FF profusion [23-26]. Indeed, median survival ranges between studies from 28.8 months [26] to 69 months [24] and to 78 months in the present one, and the scoring systems of profusion of FF are different between study groups varying from the semiquantitative methods including the Brompton, Denver, and the Michigan scores [20-22,25] to more sophisticated quantitative methods based on measurements with analytic software [23,24,26].

Our explanation for the absence of a relationship of FF profusion and indices of severity and survival observed in this study might first relate to the fact that FF, are only one, though certainly the one with a major pathogenetic role, of all the histopathologic characteristics of UIP/IPF, and [2,31] secondly, to the fact that the geographically limited (small tissue size) of the lung sampled may not necessary reflect the resultant of the histopathology derangement in individual cases. Consequently, the potential role of FF, sites of dynamic ongoing active injury in a chronic progressive interstitial lung disease as UIP/IPF is, [32] may better reflect the previously observed relationship with indexes of disease deterioration such as decrease of DLCO and FVC [21,25] and dyspnea worsening [25].

On the other hand, progressive breathlessness, the most disabling symptom of IPF though multifactorial better reflects the overall of lung damage and its related consequences on lung mechanics and gas exchange [33]. Thus, the finding of this study that the MRC dyspnea scale does not correlate with FF profusion could be explained by the fact that the defects in lung mechanics and gas exchange which manifest clinically with progressive exertional dyspnea might result from the entire combination of histological changes that characterize UIP/IPF and their geographical extension. Indeed, dyspnea scores are better shown to relate with histological parameters such as end-stage fibrosis and honeycombing [7,11].

The major weakness of the present study is its relatively small number of patients and its retrospective design. However, it includes a homogeneous population of one academic department and its retrospective design is a valid approach for the study of a disease with low prevalence such as UIP/IPF. Prospective studies require very long period of observation. In addition, the survival rate of our patients is higher than that of patients included in other studies and this might relate: firstly to the very early stage disease of patients included as shown by the fact that 19 of them presented MRC score 1 and 2 and no patient with MRC 5 was included, due to the inability to perform lung biopsy in such severely ill patients, and secondly to the fact that most patients were treatment naïve or discontinued treatment on the first sign of infection. Regarding this ultimate it is well known that UIP/IPF is unresponsive to any immunosuppressive therapy which on the contrary leads unavoidably to severe and often terminal infective complications [34].

## Conclusions

In conclusion, the missing relationship between the MRC dyspnea scale and the FF profusion could be explained with the fact that dyspnea in IPF better reflects the overall of lung damage and its related consequences on lung mechanics and gas exchange. FF, only one of its histological hallmarks, might not reflect its entire histology derangement also constrained by the geographically limited sampled lung tissue. This might be also valid for the observed lack of association between FF and survival or functional parameters.

## Competing interests

The authors declare that they have no competing interests.

## Authors' contributions

CT has collected clinical data and gathered surgical lung biopsy slides and drafted the first version of the manuscript. EDM participated in gathering and evaluating clinical information, surgical lung biopsies and in writing the manuscript. CM has reviewed and scored all surgical lung biopsies, provided Figure 2 and participated in writing parts of the manuscript. CS did the statistical analysis. LFK has participated in gathering clinical information, in interpretation of clinical and statistical information and in the critical review of the manuscript. KK has participated in gathering surgical lung biopsies, in interpretation of clinical data and in critical review of parts of the manuscript. DR reviewed and scored all surgical lung biopsies and participated in writing parts of the manuscript. SAP conceived the study, participated in its design and coordination and drafted the final version of the manuscript. All authors read and approved the final manuscript.
